# Embryonic Stem Cell Lines of Nonhuman Primates

**DOI:** 10.1100/tsw.2002.829

**Published:** 2002-06-26

**Authors:** Norio Nakatsuji, Hirofumi Suemori

**Affiliations:** Department of Development and Differentiation, Institute for Frontier Medical Sciences, Kyoto University, 53 Kawaharacho, Shogoin, Sakyo-ku, Kyoto 606-8507, Japan

**Keywords:** pluripotent stem cells, embryonic stem (ES) cell lines, EG cells, primate, cynomolgus monkey, cell therapy, regenerative medicine

## Abstract

Human embryonic stem (ES) cell lines have opened great potential and expectation for cell therapy and regenerative medicine. Monkey and human ES cell lines, which are very similar to each other, have been established from monkey blastocysts and surplus human blastocysts from fertility clinics.

Nonhuman primate ES cell lines provide important research tools for basic and applicative research. Firstly, they provide wider aspects of investigation of the regulative mechanisms of stem cells and cell differentiation among primate species. Secondly, their usage does not need clearance or permission from the regulative rules in many countries that are associated with the ethical aspects of human ES cells, although human and nonhuman embryos and fetuses are very similar to each other. Lastly and most importantly, they are indispensable for animal models of cell therapy to test effectiveness, safety, and immunological reaction of the allogenic transplantation in a setting similar to the treatment of human diseases.

So far, ES cell lines have been established from rhesus monkey (Macaca mulatta), common marmoset (Callithrix jacchus), and cynomolgus monkey (Macaca fascicularis), using blastocysts produced naturally or by in vitro fertilization (IVF) and intracytoplasmic sperm injection (ICSI). These cell lines seem to have very similar characteristics. They express alkaline phosphatase activity and stage-specific embryonic antigen (SSEA)-4 and, in most cases, SSEA-3. Their pluripotency was confirmed by the formation of embryoid bodies and differentiation into various cell types in culture and also by the formation of teratomas that contained many types of differentiated tissues including derivatives of three germ layers after transplantation into the severe combined immunodeficiency (SCID) mice.

The noneffectiveness of the leukemia inhibitory factor (LIF) signal makes culture of primate and human ES cell lines prone to undergo spontaneous differentiation and thus it is difficult to maintain these stem cell colonies. Also, these ES cells are more susceptible to various stresses, causing difficulty with subculturing using enzymatic treatment and cloning from single cells. However, with various improvements in culture methods, it is now possible to maintain stable colonies of monkey ES cells using a serum-free medium and subculturing with trypsin treatment. Under such conditions, cynomolgus monkey ES cell lines can be maintained in an undifferentiated state with a normal karyotype and pluripotency even after prolonged periods of culture over 1 year. Such progress should facilitate many aspects of stem cell research using both nonhuman primate and human ES cell lines.

## INTRODUCTION

Human pluripotent stem cell lines, which include embryonic stem (ES)[1,2] and embryonic germ (EG)[3] cell lines, have opened great potential and expectation for cell therapy and regenerative medicine, because many types of human cells can be produced by unlimited proliferation and differentiation of stem cells in culture (Fig. 1). For many years, mouse ES cell lines have been important tools for basic biology to investigate cell differentiation and development and to carry out gene targeting for analysis of gene function in mice. ES cell lines are derived from the inner cell mass (ICM) of blastocysts[4,5], while EG cell lines are derived from primordial germ cells[6,7,8] isolated from fetuses. Pluripotency of mouse ES or EG cells can be demonstrated by differentiation into various cell types in culture or in teratomas formed by transplantation into syngenic mice, and also by the production of chimeric mice, in which ES cells can contribute to all tissues and organs including the germ cell lineage.

The most important ability of human pluripotent cell lines in medical research is their unlimited proliferation in culture and subsequent differentiation into many types of functional cells. Therefore, it is most important to determine reliable methods to maintain proliferation of stem cells, and to devise various methods of inducing differentiation into particular useful cells. Already, mostly by using mouse ES cells, there have been reports of the production of various types of neurons including dopamine-producing cells[9,10], glia[11], cardiac muscle[12], hematopoietic cells, and endothelial cells[13,14,15,16]. Also, insulin-producing pancreatic cells[17] and hepatocytes will be produced in the near future. All of these cell types have important medical applications for cell therapy of corresponding diseases. There will be an increasing number of reports of production of such cells using monkey and human ES cells in the coming years.

## MOUSE, MONKEY AND HUMAN ES CELLS

In 1995, Thomson and collaborators[18,19,20] reported the establishment of ES cell lines from blastocysts of the rhesus monkey and marmoset. The former species are macaques, which are phylogenically close to humans (Fig. 2), and the latter species have an advantage of their smaller size in handling and breeding. The methods of Thomson et al.[21] for establishment of ES cell lines were almost the same as those used to derive mouse ES cell lines. For example, they used

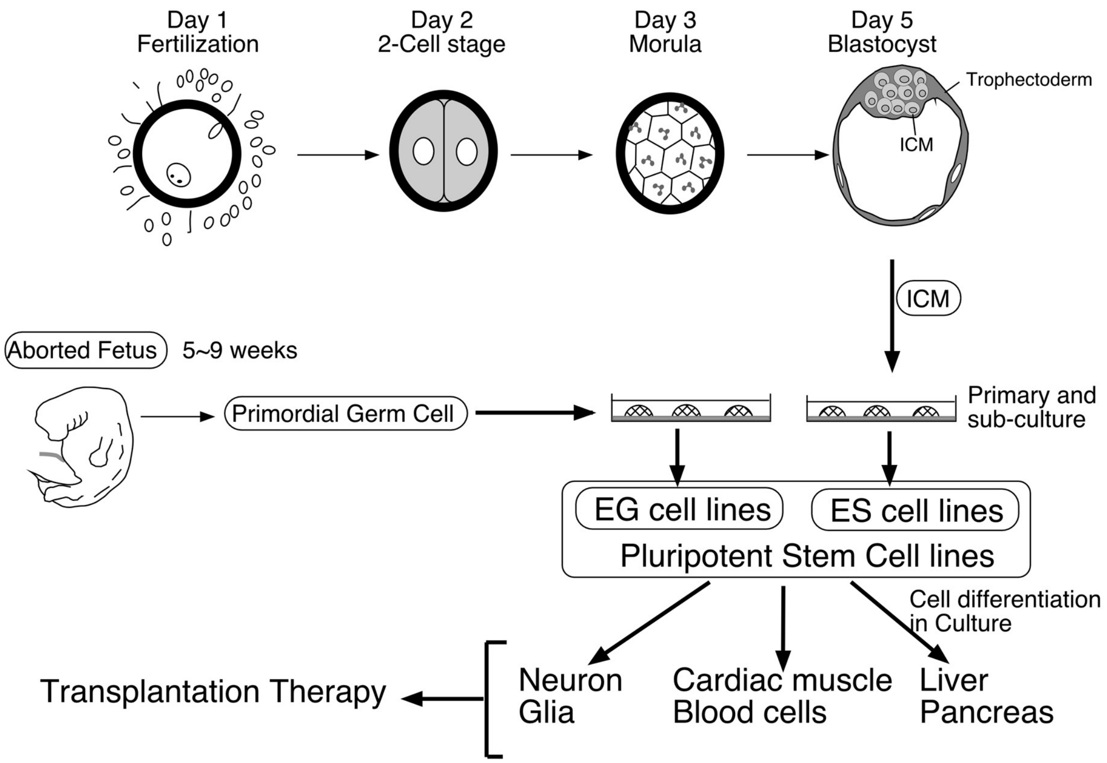

**FIGURE 1**. A diagram showing establishment of human ES cell lines from blastocysts and that of EG cell lines from fetal germ cells. Such pluripotent cell lines can be maintained and grown for a long period in culture and induced to differentiate into various cell types, which can be used for cell transplantation therapy and other purposes in medical research.

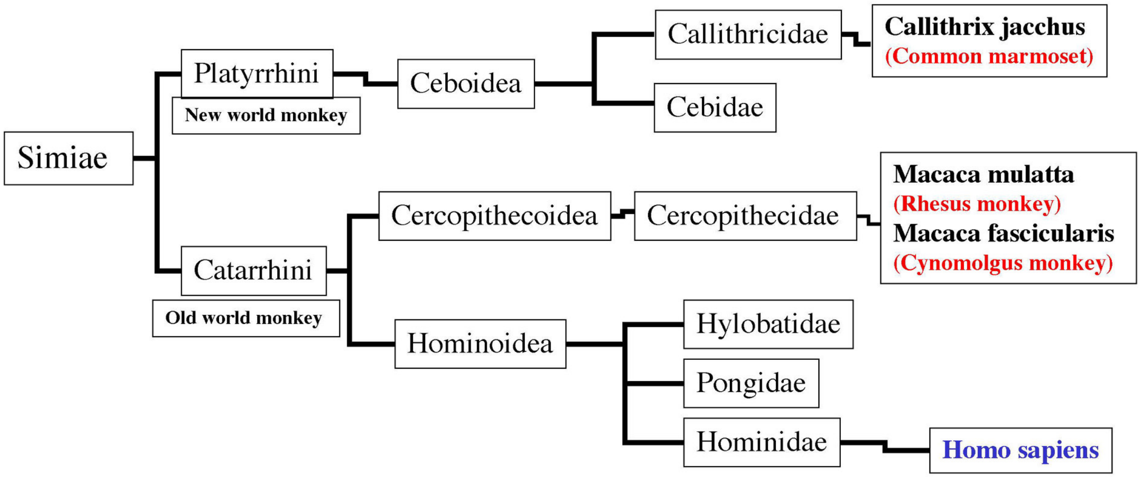

**FIGURE 2**. A phylogenic tree of primates, indicating relationship among the human, rhesus and cynomolgus monkeys, and common marmoset. Nonhuman primates closest to humans, such as chimpanzees and gorillas belonging to Hominoidea (Pongidae), are virtually unavailable for research purposes as experimental animals. Therefore, macaques are the most useful for preclinical research in regenerative medicine.

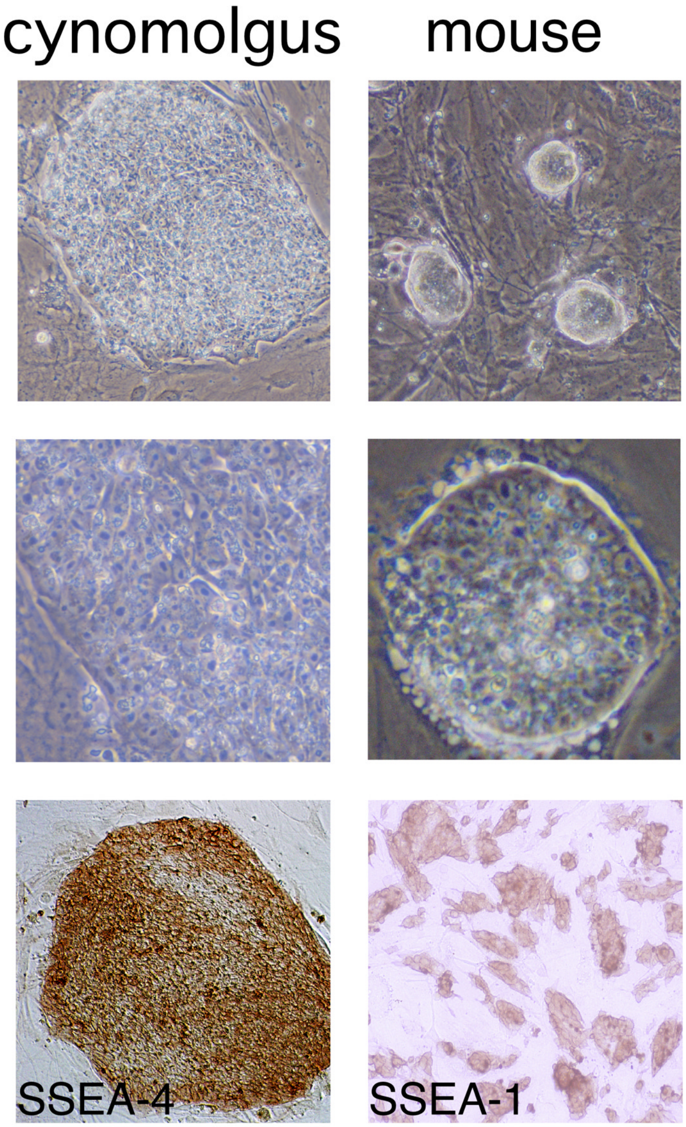

**FIGURE 3**. Morphology of cynomolgus monkey and mouse ES cells and expression of SSEA antigens. Monkey ES cells form flatter colonies than mouse ES cell colonies. Also, cell boundaries are visible in the monkey colonies, while the mouse colonies are made of ES cells compacted each other so that individual cells are unrecognizable.

mouse embryonic fibroblasts as feeder cells. However, there were a few differences between mouse and monkey ES cells themselves. Firstly, addition of LIF in the culture medium produced no effects in the maintenance of primate stem cells. Secondly, the shape of monkey ES cell colonies was flatter than the compacted and domed mouse ES cell colonies (Fig. 3). Thirdly, expression of some stem cell markers was different (Table 1). Although alkaline phosphatase activity was detected, stage-specific embryonic antigen (SSEA)-1 antigen, which is strongly expressed in mouse ES cells, was not expressed in the primate lines. Instead, SSEA-3 and SSEA-4 antigens were expressed in monkey ES cells. These ES cells exhibited pluripotency by formation of teratomas containing many types of differentiated cells and tissues when

**TABLE 1**
**Expression of Various Stem Cell Markers by Monkey ES Cells, Human ES and EG Cells, and Mouse ES and EG Cells**

| | Cynomolgus ES | Rhesus ES | Marmoset ES | Human ES | Human EG | Mouse ES, EG |
|---|---|---|---|---|---|---|
| ALP | + | + | + | + | + | + |
| SSEA-1 | – | – | – | – | + | + |
| SSEA-3 | – | + | + | +* | + | – |
| SSEA-4 | + | + | + | + | + | – |
| TRA-1-60 | ND | + | + | + | + | –** |
| Oct-3/4 | +*** | ND | ND | + | MD | + |
| References | [23] | [18,20] | [19,20] | [1,2] | [3] | |

*Note:* A summary of expression of stem cell markers including alkaline phosphatase (ALP), SSEA and TRA antigens, and Oct-3/4 gene, by ES cells of the cynomolgus monkey, rhesus monkey, common marmoset, human,and mouse. EG cells of the human and mouse are also included.

* Various expression levels among cells and colonies.

** The antibody dose did not react with mouse cells.

*** Our unpublished data.

ND: Not determined.

transplanted into immunodeficient SCID (severe combined immunodeficiency) mice, which contained not only the frequently recognizable ectoderm and mesoderm tissues but also the endoderm derivatives such as pancreas and intestine[22]. Finally, these primate ES cell lines frequently produced trophectoderm lineage as indicated by appearance of trophoblast and production of chorionic gonadotropin[18,19,20], in contrast to mouse ES cells that rarely produce trophoblast.

Then, in 1998, human ES cell lines were established from the ICM of blastocysts, which had been produced but not used in the clinical treatment of infertility[1]. Human ES cell lines showed very similar characteristics to monkey ES cells as indicated in Table 1. Since then, other groups have reported establishment of human ES cell lines[2]. On the other hand, there has been only one report, in 1998, of the establishment of human EG cell lines from primordial germ cells obtained from aborted fetuses at 5–9 weeks of pregnancy[3]. Such EG cell colonies showed different characteristics from human ES cells, which is surprising because mouse ES and EG cells are almost indistinguishable. Human EG cells made tightly compacted colonies, expressed SSEA-1 in addition to SSEA-3 and -4 antigens (Table 1). Also, the presence of LIF was effective for maintenance of human EG cells[3]. Pluripotency of these EG cell was shown by formation of embryoid bodies (EBs) containing various cell types. Thus, there are significant differences between mouse and primate ES and EG cell lines. Therefore, we started to work on primate ES cells.

## SIGNIFICANCE OF NONHUMAN PRIMATE ES CELL LINES IN PRECLINICAL RESEARCH

Before clinical application of cell therapy using human ES cells, we need to test various aspects of cell transplantation by using animal models. Nonhuman primate models are necessary in addition to rodent models for preclinical assessment because they are closer to humans in phylogeny, body sizes, and physiology. For example, the tumorgenic potential of cell transplantation must be tested using animal models before clinical application. ES or EG cells are not malignant tumor cells but they show unlimited proliferation and form benign tumors in immunorepressed animals. Therefore, removal of stem cells from differentiated cell populations is necessary before cell transplantation. In clinical application, administration of immunosuppressive drugs may be necessary to prevent allogenic rejection. Possibility of the tumorgenesis from transplanted cells should be evaluated using not only the rodent models but also monkey models which have much more physiological similarities to the human treatment in immunological responses and drug usage. To obtain higher degrees of safety, implementation of a suicidal or cell ablation gene in ES cells may be necessary to trigger cell death if something goes wrong after transplantation. Evaluation of these methodologies must be done in a closely related species before human trials are attempted.

Another important aspect to evaluate is the immunorejection after transplantation of allogenic ES-derived cells. Such immunological responses are more adequately predicted by using nonhuman primate models rather than other animal models. Such problems of cell therapy may be improved by genetical alteration of ES cell lines to reduce their antigenicity. The major histocompatibility complex (MHC) genes or HLA genes of ES cells could be altered by disruption of the endogenous gene by gene targeting. Then, such ES cell lines can be further modified by subsequent introduction of desired HLA type genes. Complete matching of the many HLA types would be impossible between the modified ES cells and patients, but matching of the major types may well reduce immunological rejection to clinically manageable levels.

In the course of such investigation, careful assessment of safety and effectiveness using proper animal models is required before clinical application to patients. Mouse ES cells are the best characterized, and many mouse models of relevant diseases are available. However, mouse ES cells differ from human ES cells in many respects. Moreover, the physiology of mice is significantly different from humans. In addition to the regulation of immunological responses and tumorgenesis, sizes and structures of various organs and tissues are very important for actual cell transplantation. For example, the brain is one of important targets of cell therapy. Macaques have the brain structures most similar to humans among various experimental animals. Also, assessment of the cell therapy of other anticipated targets such as the liver and eyes requires proper sizes of these organs to simulate transplantation methods, number of engrafted cells, and physiological effects caused by the transplanted cells. Thus, the mouse system is less suitable for preclinical research aimed at cell transplantation therapy than model systems using nonhuman primates. The rhesus and cynomolgus monkeys are macaques belonging to the old world monkeys, which are closely related to humans (Fig. 2). They are bred as experimental animals and widely used for medical research. Also, various disease models, such as Parkinson disease, of macaques are available for the research purpose. For such reasons, ES cell lines of nonhuman primates are valuable and indispensable tools for preclinical research in regenerative medicine.

## ESTABLISHMENT OF CYNOMOLGUS ES CELL LINES

We established ES cell lines from cynomolgus monkey (*Macaca fascicularis*) blastocysts produced by *in vitro* fertilization (IVF) or intracytoplasmic sperm injection (ICSI)[23]. We used cynomolgus monkeys because they are the most widely used macaques for medical and pharmaceutical research in Japan. Therefore, these cell lines could be used for studies of primate models of several human diseases. We examined expression of several stem cell markers by cynomolgus ES cells (Table 1, Fig. 3). They expressed the alkaline phosphatase activity and SSEA-4 antigen, but not SSEA-3 or SSEA-1 antigens. While human and rhesus ES cells were reported to express the SSEA-3 antigen at variable levels among stem cell colonies, cynomolgus

**TABLE 2**
**Karyotype Analysis of Two ES Cell Lines of the Cynomolgus Monkey**

| Cell Line | Passage (Month) | Normal/Counted (%) | Sex |
|---|---|---|---|
| CMK6 | 47 (6) | 14/20 (70) | Male |
| | 84 (12) | 17/25 (68) | |
| CMK9 | 18 (3) | 16/20 (80) | Female |
| | 64 (7) | 14/16 (88) | |

*Note*: Chromosome number of each cell line was counted at various passage numbers. Also shown are approximate culture periods in months. Spreads showing diploid (40 + XX or XY) chromosomes were counted as normal. Karyotypic sex is also shown.

ES cells were negative for SSEA-3 in immunostaining. A very recent study of the cynomolgus ES cell lines established from parthenogenetic embryos[24] also reported negative expression of the SSEA-3 antigen.

Cynomolgus ES cells showed extensive spontaneous differentiation when cultured using the standard medium for mouse ES cells with feeder cells and LIF. However, after improvement of culture methods described later, these monkey ES cell lines were successfully maintained in an undifferentiated state and with a normal karyotype for prolonged periods of more than 12 months (Table 2). We obtained cell lines with either the male or female karyotypes[23].

When cynomolgus ES cells were allowed to grow to higher densities to induce differentiation, several kinds of differentiated cells were observed. They included vesicular epithelia resembling the visceral endoderm or yolk sac and mesenchymal cells that showed outgrowth from cell clumps. EBs were formed by culturing ES cell aggregates in Petri dishes. Extended culture resulted in the formation of cystic EBs, and after plating onto tissue culture dishes they produced many types of differentiated cells, such as clusters of neurons and pigment cells. Such differentiation potency of the cynomolgus ES cells has been further confirmed by production of the dopaminergic neurons and retinal pigmented epithelia by using culture conditions to induce such differentiation in our very recent study[25]. We are currently planning to transplant such produced dopaminergic neurons into the brain of cynomolgus monkeys to evaluate effectiveness and safety of the cell therapy for the Parkinson disease.

To examine pluripotency more closely, we transplanted cynomolgus ES cells into SCID mice to produce teratomas (Fig. 4). Histological examination of these teratomas revealed that they contained various tissues derived from all three embryonic germ layers. We frequently observed ectodermal tissues containing neurons, glia, glands, and epithelia. Mesodermal tissues such as muscle, cartilage, and bone were also observed frequently as well as typical hair follicles. However, endodermal tissues such as gut epithelium were relatively rare. A columnar epithelium, probably tracheal ciliated epithelium, was the most frequently recognizable putative endodermal tissue. Expression of several tissue-specific proteins were examined to assess differentiation in teratomas by using antibodies prepared for pathological examination of human specimens that recognize tissue-specific antigens (Fig. 4). Most tissues expressed typical tissue-specific markers. For example, mature neurons and glia surrounding the primitive neuroectoderm expressed the neuron specific enolase (NSE). Glial cells also expressed the glial fibrilally acidic protein (GFAP). Muscle and cartilage, both of which are derived from the mesoderm, expressed specific markers desmin and S-100, respectively. As for the endodermal tissues, we found cell clusters expressing alpha-fetoprotein, but it may be expressed not only by the liver but also by the yolk sac endoderm. We did not observe cell clusters expressing insulin.

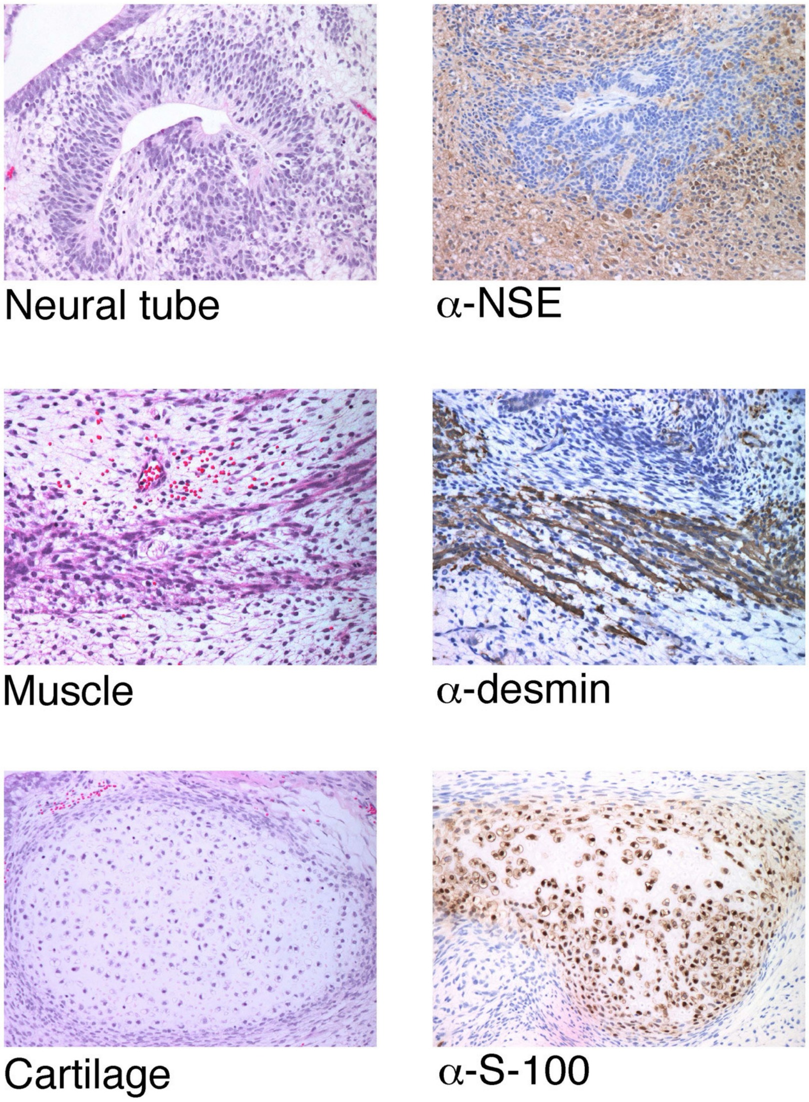

**FIGURE 4**. Differentiation of the neural tube, muscle, and cartilage in teratomas formed by hypodermal injection of cynomolgus monkey ES cells into a SCID mouse and expression of tissue specific markers. Immunohistochemistry indicated differentiation of neurons and glia expressing neuron specific enolase, muscle expressing desmin and cartilage expressing S-100.

## IMPROVED MAINTENANCE AND PROLIFERATION OF PRIMATE ES CELL LINES

Spontaneous differentiation of stem cells even in the presence of LIF and feeder cells and low efficiency in subculturing have been the major problems to hinder stable maintenance of monkey and human ES cell lines. In such conditions, it was necessary to collect individual colonies for enrichment of undifferentiated stem cells at certain intervals during maintenance of ES cell lines. Also, a relatively unreliable method of mechanical disruption of ES cell colonies was used to avoid cell damage, instead of a proteinase treatment that would enable uniform dissociation of cell colonies. Such problems must be solved before progress on various aspects of research using primate ES cells can be made. Recently, however, we have succeeded in obtaining significant improvement of these aspects in propagation of cynomolgus ES cell lines[23].

Spontaneous appearance of differentiated cells during monkey ES cell culture was reduced remarkably when fetal bovine serum (FBS) was replaced by Knockout Serum Replacement (KSR, GIBCO) in the culture medium. Although we used FBS samples that had been tested for maintenance of mouse ES cells, it is possible that such FBS still contained various differentiation-inducing factors, which were not present in the KSR. In such serum-free medium, cynomolgus ES cells were maintained in the undifferentiated state for a longer period without periodical collection of the stem cell colonies, which had been necessary when using the FBS medium. In KSR medium however, ES cells exhibited flatter morphology and slower growth rates than in the FBS medium. Even so, splitting of the ES cell culture into 3~4 duplicate dishes was possible in every 3–4 days. Addition of bFGF was reported to increase the cloning efficiency of human ES cells[26]. However, its addition to the medium had little effect on cynomolgus ES cells.

Similar to other primate ES cell lines, cynomolgus ES cells exhibited a very low plating efficiency when dissociated into single cells. During subculturing, limited dissociation into cell clusters of 10–50 stem cells was required to enable continued growth. Thus, the standard dissociation procedure for mouse ES cells using trypsin caused excessive damage to the monkey ES cells. Without trypsin however, we could not obtain proper reproducible dissociation. After testing various conditions, we devised an adequate method for efficient subculturing by using 0.25% trypsin supplemented with 1 mM $CaCl_2$ and 20% KSR. Presence of Ca ions at this concentration slowed down the cell dissociation by trypsin. Also, it may well give protection effects to the cell membrane. Thus, this method enabled well-controlled and reproducible dissociation process of the ES cell colonies throughout the whole culture dish to harvest stem cells in small aggregates of 10–50 cells for routine efficient subculturing.

## DIFFERENCES BETWEEN PRIMATE AND MOUSE ES CELLS

An important difference of primate ES cells compared with mouse ES cells is production of the trophectoderm lineage. It has been known that mouse ES cells only contribute rarely to the trophectoderm lineage in chimeric embryos[27]. This is consistent with the cell lineage pathways of early development because the ICM is formed after differentiation of the trophectoderm from still undifferentiated morula cells, and ICM cells lose the potential to form trophectoderm at the late blastocyst stage[28]. In contrast, monkey and human ES cells are reported to produce large amounts of the trophectoderm lineage and trophoblastic giant cells with chorionic gonadotropin production. Thus, primate ES cells may represent an earlier stage such as blastomeres up to the morula stage.

Among the molecular markers of stem cells, alkaline phosphatase activity is always present in all primate and mouse ES and EG cells. However, the SSEA antigens show interesting differences in expression (Table 1). The SSEA-1 antigen is expressed in mouse ES and EG cells as well as human EG cells, but not in monkey and human ES cells. On the other hand, the SSEA-4 antigen is expressed in monkey ES cells and human ES and EG cells, but not in mouse ES cells. The expression pattern of the SSEA-3 antigen is similar to SSEA-4 but at variable levels among the stem cell colonies, and nondetectable in cynomolgus ES cells. Interestingly, previous studies[29,30] reported that SSEA-3 and SSEA–4 antigens are expressed in mouse preimplantation embryos at cleavage stages earlier than the SSEA-1 antigen. Also, human ES and EC cells show transient expression of the SSEA-1 antigen during differentiation from stem cells[1,31].

These various facts suggest that primate ES cells may represent an earlier developmental stage, such as the cleavage or morula stage, than mouse ES cells, which represent the ICM or epiblast of the early postimplantation embryo. If so, human EG cells could be positioned between these two stages. Thus, primate ES cells may have undergone dedifferentiation from the ICM stage in culture. We need more data to elucidate such speculation, but these aspects may lead to interesting insights as to which pluripotent stage is most stable for early embryonic cells of various mammalian species.

## FUTURE GOALS OF RESEARCH USING PRIMATE ES CELLS

For further progress in basic research and the medical application of primate and human ES cells, we still need to improve many aspects of the manipulation of stem cells. Firstly, it is necessary to propagate ES cells in a completely defined medium, excluding not only the serum but also animal proteins for clinical application. Secondly, we must find out a way to derive and maintain ES cell lines without a feeder cell layer to avoid unexpected contamination such as by animal retrovirus. To this end, there have been only incomplete trials using human ES cells[32], which reports effectiveness of coating of the culture substratum with extracellular matrices and addition of conditioned medium made from mouse feeder cells. Thirdly, and most importantly, we need to devise best methods for gene targeting to produce genetically modified monkey and human ES cells. There have been only a few studies of gene transfection in these lines[33, our unpublished results]. Such genetical modifications would enable reduction of antigenicity in cell therapy and efficient selection of particularly useful cell types.

In every aspect of the ES cell study, nonhuman primate ES cell lines provide important research tools for basic and applicative research. In most countries, usage of human ES cells is strictly regulated because of ethical problems. In such situations, monkey ES cells could provide invaluable materials for research to advance various aspects of regenerative medicine directly applicable to humans. Finally, they are indispensable for preclinical research using primate models of allogenic cell transplantation therapy to evaluate effectiveness, safety, and immunological reaction in physiological conditions similar to the treatment of human diseases.

## ACKNOWLEDGEMENT

Our original study included in this review was supported in part by a Research for the Future (RFTF) program of the Japan Society for the Promotion of Science.
